# Enrichment and Training Improve Cognition in Rats with Cortical Malformations

**DOI:** 10.1371/journal.pone.0084492

**Published:** 2013-12-17

**Authors:** Kyle R. Jenks, Marcella M. Lucas, Ben A. Duffy, Ashlee A. Robbins, Barjor Gimi, Jeremy M. Barry, Rod C. Scott

**Affiliations:** 1 Department of Neurology, Geisel School of Medicine at Dartmouth, Lebanon, New Hampshire, United States of America; 2 Centre for Advanced Biomedical Imaging, Division of Medicine, University College London, London, United Kingdom; 3 Department of Radiology, Geisel School of Medicine at Dartmouth, Lebanon, New Hampshire, United States of America; 4 University College London, Institute of Child Health, London, United Kingdom; Alexander Fleming Biomedical Sciences Research Center, Greece

## Abstract

Children with malformations of cortical development (MCD) frequently have associated cognitive impairments which reduce quality of life. We hypothesized that cognitive deficits associated with MCD can be improved with environmental manipulation or additional training. The E17 methylazoxymethanol acetate (MAM) exposure model bears many anatomical hallmarks seen in human MCDs as well as similar behavioral and cognitive deficits. We divided control and MAM exposed Sprague-Dawley rats into enriched and non-enriched groups and tested performance in the Morris water maze. Another group similarly divided underwent sociability testing and also underwent Magnetic Resonance Imaging (MRI) scans pre and post enrichment. A third group of control and MAM rats without enrichment were trained until they reached criterion on the place avoidance task. MAM rats had impaired performance on spatial tasks and enrichment improved performance of both control and MAM animals. Although MAM rats did not have a deficit in sociability they showed similar improvement with enrichment as controls. MRI revealed a whole brain volume decrease with MAM exposure, and an increase in both MAM and control enriched volumes in comparison to non-enriched animals. In the place avoidance task, MAM rats required approximately 3 times as long to reach criterion as control animals, but with additional training were able to reach control performance. Environmental manipulation and additional training can improve cognition in a rodent MCD model. We therefore suggest that patients with MCD may benefit from appropriate alterations in educational strategies, social interaction and environment. These factors should be considered in therapeutic strategies.

## Introduction

Malformations of Cortical Development (MCD) are a wide range of disorders with many genetic and environmental causes [1,2]. Clinically, MCD is a term used to group together disruptions in brain development caused by a failure in neuronal migration or neuronal development. MCD’s are frequently associated with epilepsy, developmental delay, and psychiatric impairments such as schizophrenia [3]. 

In addition to cognitive effects, there is also an important link between MCD and childhood epilepsy. Forty percent of children with refractory epilepsy are estimated to have an underlying cortical malformation [4]. Children with MCD and epilepsy face a potentially crippling additive effect wherein both conditions simultaneously and independently contribute to developmental delay and cognitive impairment. The underlying MCD leads to potential developmental delay and cognitive impairment due to anatomical changes, while epilepsy during childhood is associated with less time spent in school, restricted activities, and behavioral disorders [5-9] possibly due to a lack of specialized care amongst the parents, teachers and physicians involved in caring for children with epilepsy [10,11]. This could be considered a type of environmental deprivation and may have a negative impact on cognitive and behavioral outcomes. 

Even though it is not possible to restore brain structures that are malformed during development, it may be possible to attenuate the cognitive deficits associated with MCD through specialized care. We hypothesize that modifying environments and improving educational strategies will be effective in alleviating some of the deficits seen in children with MCD. Previous work over the last 60 years has shown that animals raised in enriched environments and in social groups (rather than in isolation) have remarkable improvements in adult neurogenesis, dendritic complexity, synaptogenesis, angiogenesis, growth factors, long term potentiation, hippocampal volume, brain volume and spatial cognition [12-16]. In addition, an enriched environment has been shown to be neuroprotective against cerebral injury, kainate induced seizures, and excitotoxicity [17]. However, most of these experiments have been carried out in animals with normally structured brains and it remains uncertain whether environmental manipulation will have beneficial effects in the context of a malformed brain. 

Prior to performing extremely difficult and complex studies in humans it is important to establish that environmental and training manipulations can alter behavior and brain structure in a rodent model. An established rodent model of MCD is the embryonic day (E)17 methylazoxymethanol acetate (MAM) exposure model. MAM is a potent DNA methylating agent which selectively inhibits division of mitotic cells. When injected into pregnant rats on E17, there is a well characterized disruption to the development of paralimbic, frontal and temporal cortices, and an overall decrease in hippocampal size and brain weights [18,19]. This is accompanied by a suite of cognitive impairments including deficits in attention shifting, reversal learning, object recognition, latent inhibition, and spatial cognition [18-22]. While this model of MCD does not develop spontaneous epilepsy, there is a decrease in seizure threshold and faster kindling than in controls [23]. Due to the similarities between this model and human MCD, our aim was to demonstrate that enrichment and educational strategies could have a positive outcome in an already compromised brain using the MAM model. Additionally, recent research has suggested that many cases of schizophrenia can be traced to developmental disturbances, either genetic or external in origin [20]. The MAM E17 model is used as a developmental schizophrenia model due to the common anatomical features and cognitive deficits it shares with many in the schizophrenic population [18], including decreased size of the hippocampus and cortical regions, and deficits in executive function, memory, language and attention. There exists a psychiatric interest in methods of improving cognition in this patient population apart from pharmacological treatment. 

In a series of experiments, we show that MAM treated animals are impaired in two spatial tasks in comparison to controls, that enrichment improved spatial cognition and that additional training allows animals with MCD to reach control levels of behavior on a spatial task. We also hypothesized that, due to the disrupted prefrontal cortical development seen in MAM animals, there might be a deficit in social behavior. While we did not show a deficit, we did demonstrate that MAM animals do improve their social behavior when raised in enriched environments, or alternatively interpreted; their social behavior suffers when raised in non-enriched environments. We also show that enrichment increases brain volume and hippocampal volume in both control and MAM animals. 

## Methods

### Animals

All experiments were done in accordance with National Institute of Health guidelines, and were approved by the Institutional Animal Care and Use Committee of Dartmouth Medical School and housed in conditions approved by the Association for the Assessment and Accreditation of Laboratory Animal Care International. Sprague-Dawley rats (Charles River) were housed in a 12 hour light/12 hour dark cycle with *ad libitum* access to food and water.

### Methylazomxymethanol Acetate Exposure

Methylazoxymethanol acetate was procured through the Midwest Research Institute Global (MRI Global). Pregnant dams were ordered in pairs to arrive at E16 of gestation and allowed to acclimate in the animal care facility. The following day at E17, dams were weighed. The heavier dam was always chosen to receive a MAM injection while the smaller would receive a saline injection. MAM was dissolved in sterile, injectable grade saline, and the dam was injected at 20 mg/kg intraperitoneally at volumes no greater than 0.5 ml. The other dam received an equal volume of sterile injectable grade saline. Pregnancy was allowed to proceed as normal. Dams were euthanized after pup weaning. 

### Enrichment

For the Morris water maze experiment, immediately post weaning nine MAM animals (prenatally MAM exposed) and eight control (saline exposed) animals were placed in standard, isolated housing with no objects (housing dimensions 25x48 cm). Concurrently, five control and seven MAM animals were placed in enriched housing, defined as a larger cage (38x48 cm), frequently rotated objects (edible cardboard housing, plastic dog toys), and 2-3 other cage mates of the same gender (mixed groups of MAM and control rats were identified by ear punch). Animals were left alone until testing at five months of age. For the sociability task, the same guidelines were used. 6 MAM and 5 control animals were enriched, and 6 MAM and 5 control animals were housed in standard setting (non-enriched).

### Morris Water Maze

**Figure 1:**
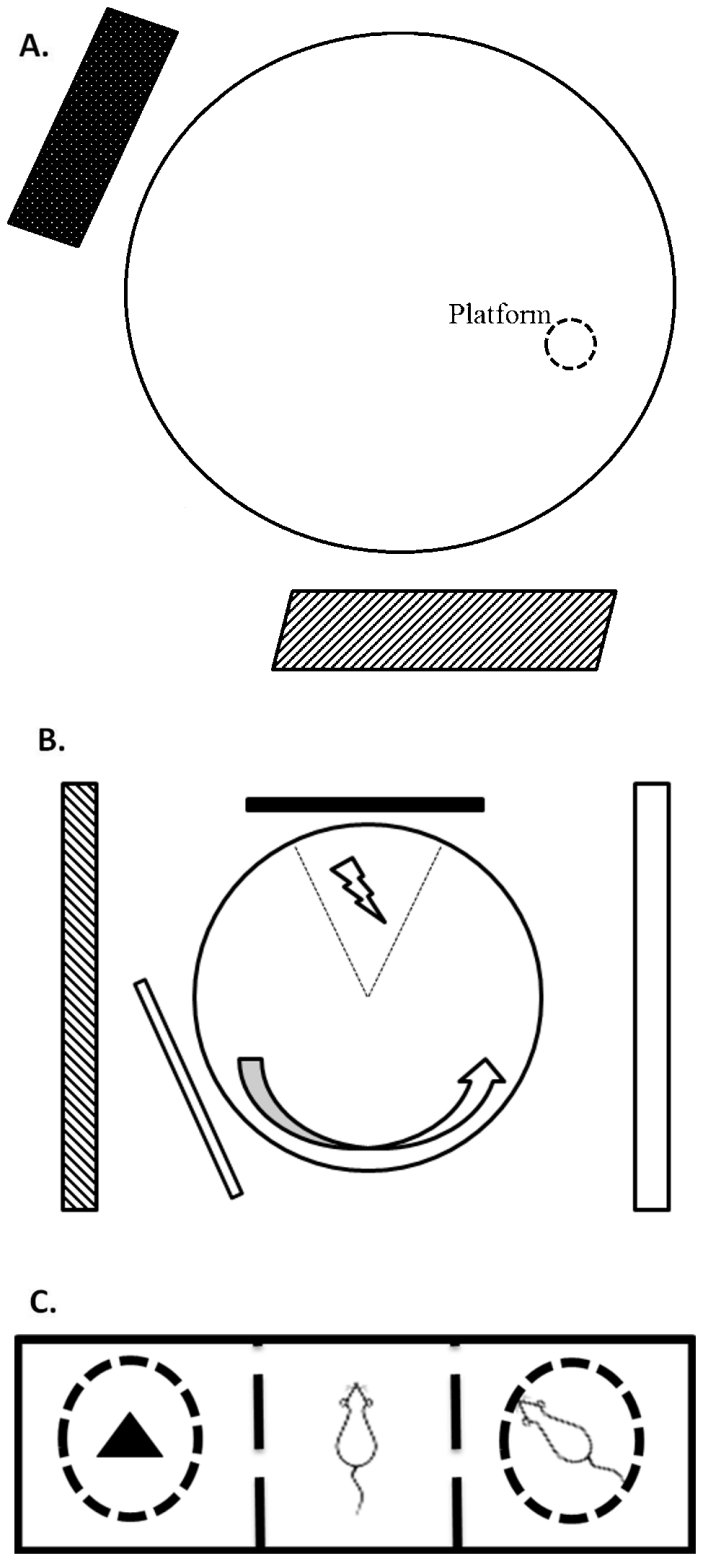
Diagrams of the behavioral set ups used in this manuscript. (**A**) Morris water maze. (**B**) Place avoidance arena. (**C**) Three chambered social choice test.

For the Morris water maze task, a water tank (diameter 2 meters, height 0.5 meters) was housed in an isolated room with the wall covered in black cloth. The tank was partially filled with water colored with non-toxic acrylic white paint to obscure a clear, 10 cm diameter plexiglass escape platform 1.5 cm below the water’s surface. Behavior was recorded from above using an analog camera connected to a DVD recorder. Two white cue cards were placed on the black curtain walls with different patterns at approximately 135 degrees in relation to one another. The platform was positioned so as not to be directly in front of either cue (Figure 1). Overhead lights were turned off to reduce glare and the room was lit by 4 spotlights positioned facing the curtains. Light levels were low, and kept set at the same level every day. Rats were placed in the room for one hour prior to experimentation to allow vision to adapt to ambient light levels. The day prior to testing, rats were lightly anesthetized with isoflurane to allow their heads to be shaved and colored with non-toxic black marker to allow camera tracking and later analysis using AnyMaze (Version 4.63, Stoelting, MA). Prior to the start of testing each following day, the rat’s head was recolored to prevent fading.

Rats were first given a 2 minute habituation session on day one. Without a platform in the tank, the rats swam for a full two minutes before being removed and dried prior to return to the home cage. Three hours later, the platform was placed in the tank. The rat was placed and held on the platform for thirty seconds prior to the trial start. Each rat was placed in the tank four times at four different entry points around the tank circumference during each trial. The rat was allowed to swim until it found the platform or two minutes elapsed, at which point it was guided to or placed on the platform. The rat was then allowed to rest on the platform for 30 seconds prior to the next entry. If necessary, the rat was held on the platform for 30 seconds. At the end of the fourth entry, due to finding the platform or elapsed two minutes, the rat was removed from the tank, dried, and returned to the home cage. The rats underwent one trial per day for four days, with entry points randomized each day.

### Active Place Avoidance

The place avoidance arena (Biosignal, Brooklyn, New York) is used for measurements of spatial cognition and memory [24-26] in which rats must avoid a pre-determined zone on a platform or receive a mild electrical shock. The day prior to the start of testing, rats were briefly anesthetized with isoflurane and implanted with a stainless steel swivel in the skin between the shoulders. This allowed attachment of a cable with an LED at the end that allowed for automated tracking and also the delivery of shock. The rat was alone in the room where testing is done, with overhead lighting facing the walls as well as lighting from below. There were cue cards on three walls, as well as two local cue cards, one black (Color-Aid 9.5 gray) and one white (Color-Aid 2.5 grey), placed on rods close to the arena but out of reach of the rat, and approximately 135 degrees in relation to each other from the arena center. The arena was a steel disc 82 cm in diameter. On day one, the animal was connected to the cable and allowed to explore the arena for 10 minutes. The number of times the animals leaned out to investigate the two proximal cues were counted. On the second day, a clear plexiglass barrier was placed around the perimeter of the arena and the arena began to spin at one revolution per minute. The rat again explored for 10 minutes. On all subsequent days the arena rotated at one revolution per minute during testing, and there was a 60 degree arc on the arena directly in front of one of the black local cue that was referred to as the shock zone. The shock zone was not visible to the rat but was stable in the room frame while the arena rotates. The rat needed to use the stationary room cues to determine where it was located in space in relation to the shock zone, and when it needed to move to avoid being brought into the shock zone by the rotation of the arena. When the rat entered the shock zone, a two milliamp shock of duration 0.5 seconds was delivered and delivered again every two seconds the rat remained in the shock zone. Training was continued with two 10 minute sessions per day until the rat met the criteria of less than five shocks within two consecutive sessions. 

After the rat had met criteria, a probe was conducted in which the local cue card locations were switched. This was to test whether the animal was relying on a spatial strategy, or associating the black cue card with shock. No shock was administered for the probe test. AnyMaze (version 4.63, Stoelting, MA) was used to count nose pokes during habituation and Biosignal software was used to make dwell time maps in which the rat’s time spent in different pixels of the environment is represented by a greyscale in which black represents no time spent and white represents most time spent. 

### Three Chambered Social Choice Test

The three chambered social choice test is commonly used to look for antisocial behavior, defined as a lack of interest in other individuals, in many autistic phenotypes [27]. Given that studies of autistic children using MRI have shown a high prevalence of MCD disorders, we decided to use sociability of a measure of prefrontal cortical mediated behavior [28,29]. In brief, a large rectangular chamber was divided into three 41 x 41 cm chambers. The walls between the chambers had openings in them sealed with plexiglass doors. A novel, gender matched rat was placed under a circular, metal grill cage (diameter 18 cm) in one end chamber, while an object was placed under an identical metal cage in the other end chamber. The rat to be tested was placed in the center chamber and allowed to acclimate for five minutes. After five minutes the two doors were lifted and the rat was allowed to freely explore for 10 minutes. Time spent in the chamber with the rat versus the chamber with the object was calculated. Analysis was performed using AnyMaze (version 4.63, Stoelting, MA).

### Magnetic Resonance Imaging (MRI)

Rats were scanned at postnatal day (P)22 prior to weaning and separation into enriched and non-enriched categories. Rats were again scanned at P150. MRI was performed using a 9.4 Tesla DirectDrive VNMRS horizontal bore system with a shielded gradient system (Agilent technologies, Palo Alto, CA) and a 4-channel rat head phased-array coil (Rapid Biomedical GmbH, Würzburg, Germany). T2 weighted imaging was performed using a multi-slice fast spin-echo (fse) sequence with the following parameters: TR = 3 s, TEeff=37 ms, ETL = 8, kzero = 9, FOV = 30 x 30 mm^2^, matrix = 256 x 256, slice thickness = 0.6 mm, inter-slice gap = 0.1 mm. The inter-slice gap was added to the voxel size in the z direction. Linear interpolation was performed in the z direction to give a final voxel size of 117 x 117 x 350 µm3. Hippocampal volume measurements were performed using manual segmentation in ITK-SNAP [30] by an operator blinded to the group identities. Whole-brain volume measurements were performed using a multi-atlas approach, in which 6 previously acquired manually segmented 3-dimensional fse datasets were co-registered to the target images using the NiftyReg affine registration algorithm [31]. Label fusion was performed using majority voting.

### Statistical Analysis

The Morris water maze experiments were evaluated using a Multivariable Cox regression time to event approach in STATA Intercooled (10.0; StataCorp, Texas). Frailty models were applied in order to deal with repeated measures. This approach allowed us to investigate the interactions between brain malformations and environmental enrichment while accounting for within animal correlations [19]. The event was defined as finding the platform and the time to event was defined as the time in milliseconds from placing the animal in the tank to the animal finding the platform. The independent predictor variables tested included day of testing, environmental enrichment, MAM, and swimming speed. 

Other parameters ascertained from the Morris water maze experiments included path efficiency and number of body rotations. These were investigated using generalized estimating equations in PASW (18.0 Chicago, Ill). The path efficiency data had a gamma distribution and the body rotation data are counts and had a Poisson distribution. The same dependent variables as for the Cox analyses were tested. 

The place avoidance experiment was evaluated using generalized estimating equations in PASW (18.0 Chicago, III). The number of entrances and number of shocks were counts and a negative binomial distribution was found to be the most appropriate. The latency to first entrance, time in zone opposite of shock zone and time in zone closest to shock zone had gamma distributions. A generalized linear model was used to evaluate the number of nose pokes and time spent nose poking each cue.

The three chambered social choice test was analyzed using SPSS Statistics (IBM, version 19). We used generalized estimating equations with enrichment and MAM treatment as factors for the dependent variables of time spent in the chamber with the novel rat, time spent in the chamber with the novel object, time spent in the center chamber, and (time spent in the chamber with the novel rat)/(time spent in the chamber with the novel object). We then entered time spent in the center chamber as a covariate for time spent in the chamber with the novel rat, time spent in the chamber with the novel object, and (time spent in the chamber with the novel rat)/(time spent in the chamber with the novel object).

MRI data was analyzed using SPSS Statistics (IBM, version 19) using a generalized linear model fitted to a gamma distribution, with MAM treatment, enrichment, and gender as factors. The change between pre and post enrichment scan was analyzed using a generalized estimating equation with a gamma distribution. 

## Results

### Morris Water Maze

**Figure 2:**
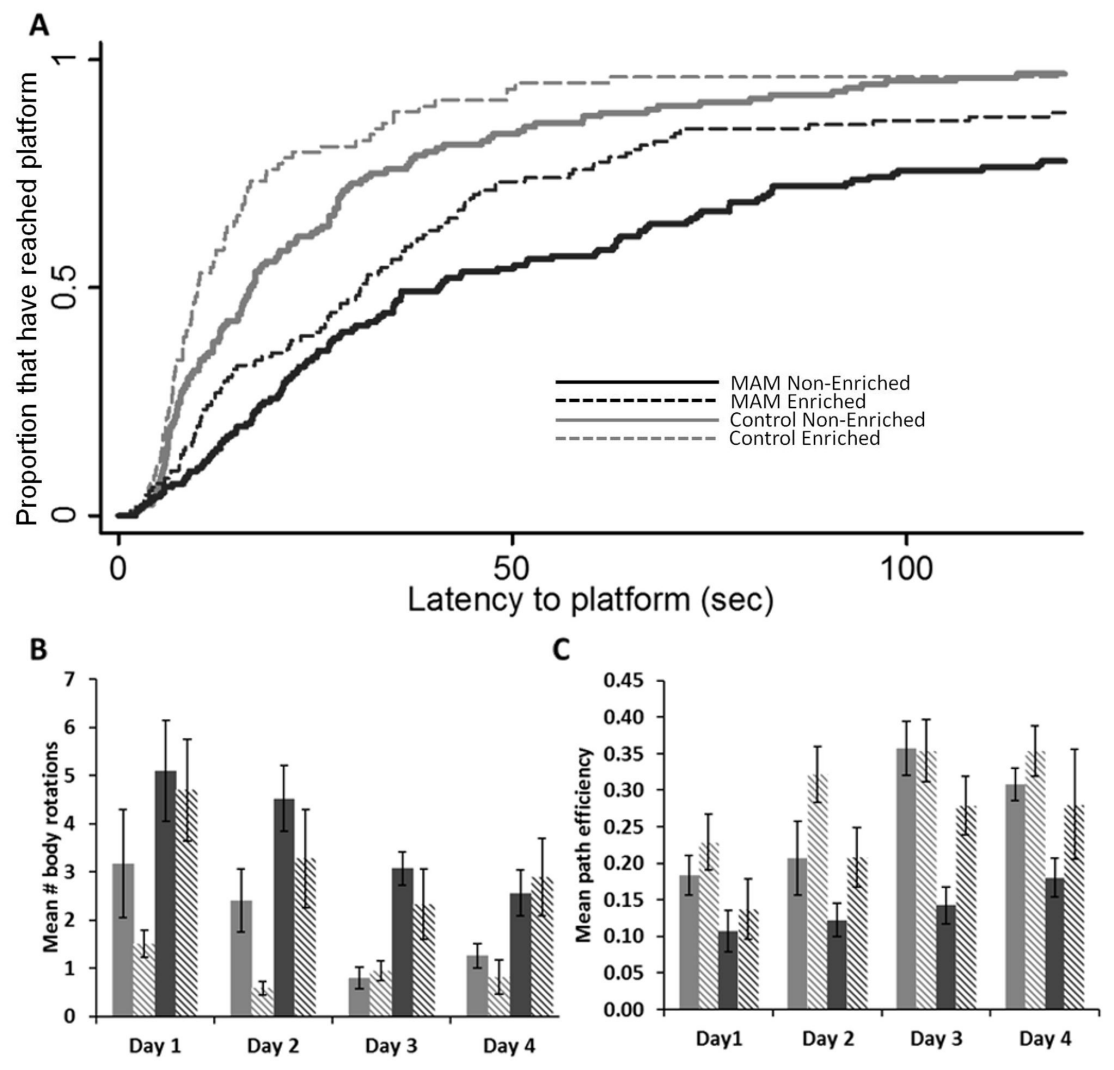
Morris water maze performance data for control and MAM treated rats. (**A**) Survival analysis representing the fraction of animals that have found the platform during a trial, capped a 120 seconds. The effect of day has been modeled in. The solid black line represents MAM non-enriched, the dotted black line represents MAM enriched, the solid grey line represents control non-enriched, and the dotted grey line represents control enriched. Controls were shown to outperform MAM animals (p<0.001), and enriched animals outperformed their non-enriched counterparts (p<0.026). (**B**) Mean number of body rotations, averaged per day. Higher numbers of rotations indicate less certainty in the where the platform is located. The solid grey bar is control non-enriched, the diagonally segmented grey bar is control enriched, the black solid bar is MAM non-enriched and the diagonally segmented black bar is MAM enriched. Controls had fewer body rotations than MAM (p<0.001), and enriched animals had fewer rotations than non-enriched (p<0.024). (**C**) Mean path efficiency per day. Path efficiency indicated the directness of the animal’s path to the platform, higher values representing a more direct path. Controls outperformed MAM (p<0.001), and enriched animals outperformed non-enriched (p<0.007).

For the Morris water maze, two litters of MAM and two litters of control were used. There were 13 total control animals, eight were non-enriched and five were enriched. There were 16 total MAM treated animals, nine non-enriched and seven enriched. All animals tested showed a higher likelihood to find the platform in later days of testing (p<0.05). Compared to day one of testing, animals were more likely to find the platform on day two (p=0.003), day three (p<0.001), and on day four (p<0.001). There was no difference in the rate of improvement between groups. After adjustment for day of testing, the time it took an animal to find the platform was significantly altered by whether the animal was MAM or control (p<0.001) (Figure 2A), whether the animal was enriched or non-enriched (p=0.026), and the animals swim speed (p=0.011).

We also investigated two variables which can be used to judge how well a subject knows the location of the platform (Figures 2B and 2C). Body rotation is a measure of how many times a subject's body completed an entire rotation of 360 degrees. Animals who knew where the platform was would be expected to make fewer rotations. Path efficiency is a measure of how direct an animal’s path is to get from their starting point to the platform. A value of one indicates perfect efficiency (straight line) while lower values would indicate less than perfect efficiency. Overall, there was a decrease of body rotations over days. After adjustment for day of testing MAM treated rats had more body rotations than controls (p<0.001), and enriched animals had fewer body rotations that their non-enriched counterparts (p=0.024). The same holds true for path efficiency, with a global increase over days (p<0.001), controls out performing MAM (p<0.001), and enriched animals out performing non-enriched animals (p=0.007). Both body rotations and path efficiency were predicted by speed (p=0.003; p<0.001 respectively).

### Active Place Avoidance

**Figure 3:**
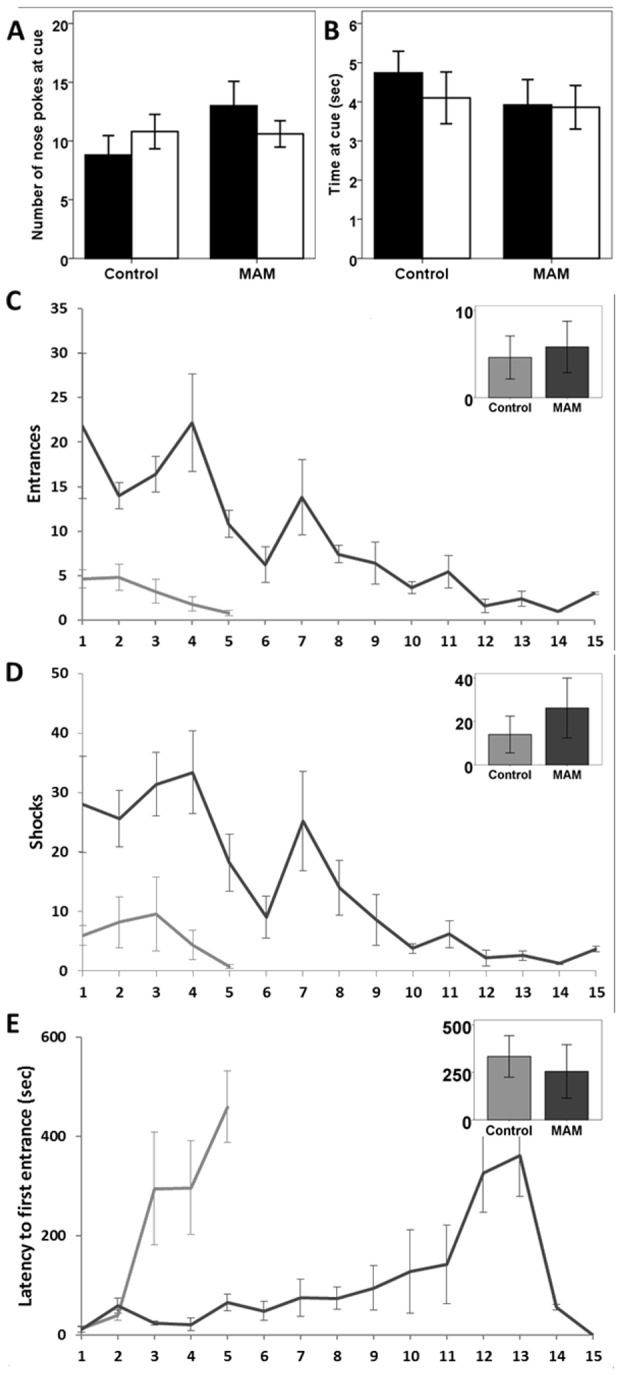
Control and MAM treated animal’s data from the place avoidance task. Control and MAM had similar levels of exploration of the two local cues (white and black) as measured by (**A**) nose pokes and (**B**) time spent at the cue. The controls (grey line) decrease entrances into the zone (**C**) and number of shocks (**D**) far more quickly than MAM (black line), but both decreased the number of entrances (p<0.001) and shocks (p<0.001) over time. (**E**) Latency to first entrance is a measure of how long an animal takes to enter the shock zone from the start of the session. MAM has significantly shorter latencies (p<0.001). Inset graphs show number of entrances, shocks, and latency for the probe session. In the probe, the black and white local cues were transposed and the shock was turned off, but entries and “shocks” that would have been delivered were still counted. This allowed us to infer the rat's strategy toward shock avoidance. Avoidance of the original shock quadrant would indicate place learning, where the location of the shock zone is defined by a configuration of multiple cues. Avoidance of the black cue would indicate stimulus-response learning, where the black cue card was associated with shock. Control and MAM both largely avoided the original shock zone rather than the black local cue.

The active place avoidance task is a measure of spatial cognition that requires an animal to constantly be alert and move to avoid a mild foot shock. There were five MAM treated rats and five control rats tested. Nose pokes at proximal cues (p=0.146 black, p=0.913 white) (Figure 3A) and time spent at cues (p=0.215 black, p=0.739 white) (Figure 3B) did not differ between groups, suggesting similar levels of exploration and interest in room cues. During the testing phase, all animals decreased the number of entrances (p<0.001) (Figure 3C) and shocks per session (p<0.001) (Figure 3D), indicating that both groups could learn the task. MAM animals took significantly more sessions (mean=6.35, 95% CI 5.47-7.22) to reach criterion (<5 shocks in 2 consecutive sessions) than controls (mean=2.52, 95% CI 1.71-3.33, p<0.001). Latency to first entrance is a measure of how long it takes following the start of a trial for an animal to enter the shock zone. As animals learn where the shock zone is located, this number is expected to increase. Across sessions, animals increased their latency to first entrance (p<0.001) (Figure 3E). MAM treated rats had significantly shorter latencies than control rats across sessions, on the fifth day of testing control rats avoided the shock zone on average 400 seconds longer than MAM animals before their first shock (p<0.001). 

**Figure 4:**
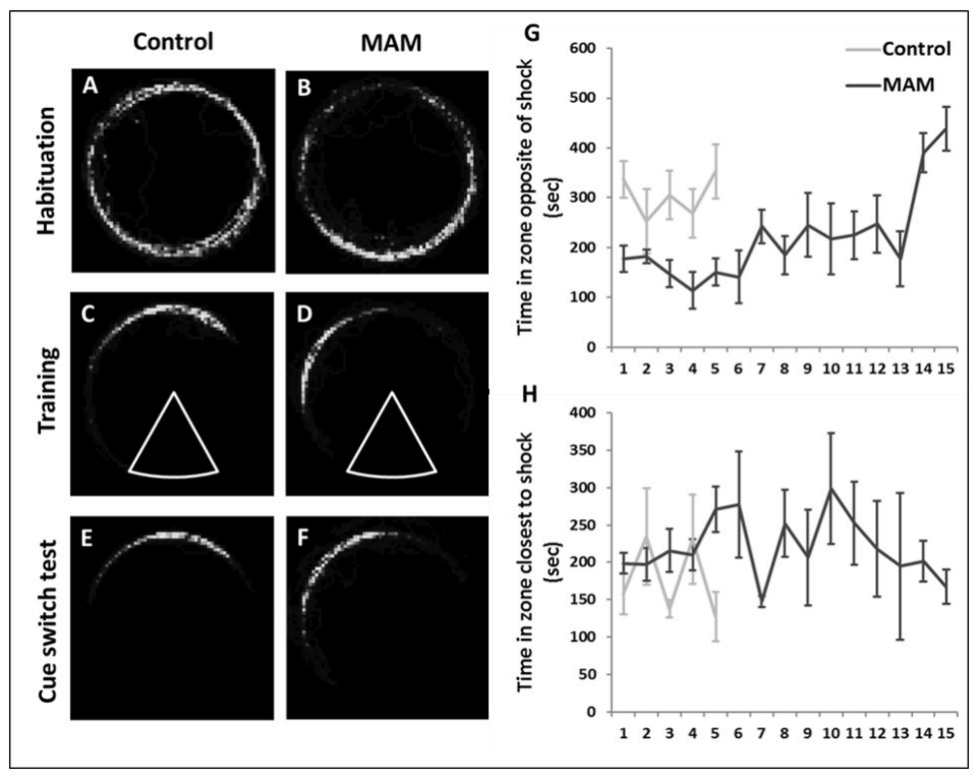
Analysis of the movement of control and MAM rats during the place avoidance test. **A-F** are dwell-time maps for individual animals where lighter colors indicate more time spent in that area. During habituation, both control (**A**) and MAM (**B**) explored the perimeter of the arena. In the sessions in which the animals met criterion, controls (**C**) avoided in the region directly opposite the shock zone (arc). MAM (**D**) avoided to the left of the shock zone where there was increased risk of shock. (**E**-**F**) The avoidance behavior did not change when the local cues where transposed. Control animals spent more time in the zone opposite the shock zone (p<0.001(**G**)), while MAM animals spent more time in the zone closest to the shock zone (left) (p<0.001(**H**)) throughout the sessions.

To understand the strategies being used to avoid the shock zone we sub-divided the arena area outside the shock zone into three zones: right of the shock zone, across from the shock zone, and left of the shock zone. The arena rotated counterclockwise, making the left zone the closest to the shock zone. Avoidance in the area opposite the shock zone would be the optimal strategy as it would decrease the probability of shock zone entrance. Control rats spent more time in the zone opposite the shock zone than MAM animals (p<0.001) (Figure 4G), while MAM animals spent more time than controls in the zone closest to the shock (session*group interaction p<0.001) (Figure 4H), indicating the use of a less effective strategy. Regardless, all individuals tested reached criteria with repeated training. 

In the dwell time maps (Figure 4A-F), we observed that both MAM and control had similar exploration in the habituation session. In the training sessions, we saw that control rats spent the majority of their time in the zone farthest from the shock zone, while MAM rats spent time in the zone closest to the shock. In the cue switch probe, MAM and control rats did not change their avoidance strategies from the last avoidance trial, indicating that both groups could navigate using the distal room cues despite the local cues being moved. 

### Three Chambered Social Choice Test

**Figure 5:**
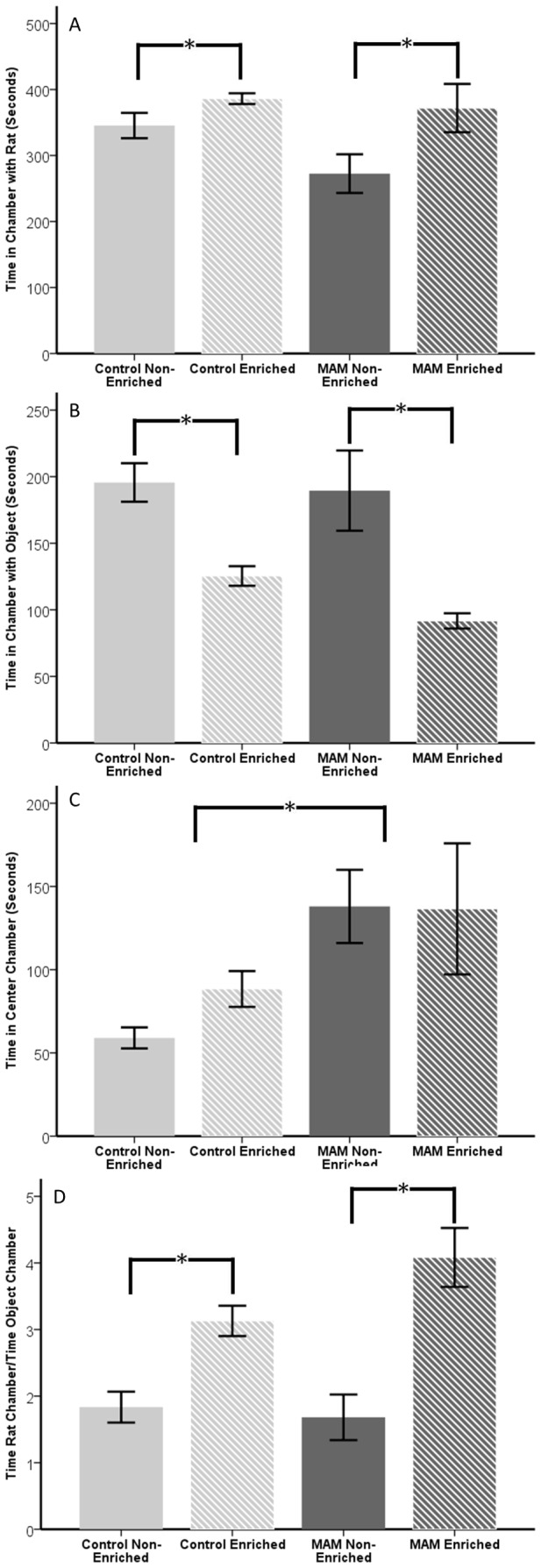
Data from the three chambered social choice test with control non-enriched, control enriched, MAM non-enriched and MAM enriched. (**A**) Control non-enriched (solid grey bar) and MAM non-enriched (solid black bar) spent less time with the novel rat than their enriched counterparts (striped bars) (p<0.006). (**B**) Non-enriched animals spent more time with the novel object than enriched animals (p<0.001). (**C**) While MAM did not significantly affect time spent in the chamber with the novel rat or novel object, there was a significant effect on time spent in the center chamber (p<0.001). (**D**) When the data was examined as a ratio of time spent with novel rat over time spent with novel object, the enrichment effect remained and the trend for a MAM effect was gone. Error bars represent standard error.

The three chambered social choice test looks for sociability deficits by giving a test animal freedom to choose between spending time in a chamber with a novel rat and spending time in a chamber with a novel object. 10 control animals were used, five non-enriched and five enriched, while 12 MAM animals were used, six non-enriched and six enriched. When we examined time spent with the novel rat, enriched animals spent more time with the novel rat than their non-enriched counterparts (p<0.006), while there was a trend for the effect of MAM to be significant (p=0.066) (Figure 5A). However, if we examined the time that the rats spent in the center chamber (in effect, not exploring either novel choice), we saw no effect of enrichment but a significant effect of MAM (p<0.001) (Figure 5C). When we took the time spent in the center as a covariate in our model of time spent in the chamber with the novel rat, we again saw that the effect of enrichment was significant (p<0.001), but the trend towards MAM being significant was gone (p=0.676).

In order to better account for the disparity in the time spent in the center, we decided to examine the ratio of time the rat spent in the chamber with the novel rat to the time spent in the chamber with the novel object. Enriched animals had a larger ratio indicating more time spent with the novel rat than the novel object (p<0.001), while MAM was not significant (p=0.491) (Figure 5D). From this we surmised that although MAM affects the time spent exploring novelty, it did not affect the proportion of time a rat chose to explore another rat over an object. Enrichment in both groups increased both the time spent exploring the chamber with the novel rat, and the ratio of time spent exploring the chamber with the novel rat over the time spent exploring the chamber with the novel object. 

### MRI

MRI was used to detect changes between groups in total brain volume and hippocampal volume. For pre enrichment, 10 control animals were scanned, five subsequently non-enriched and five subsequently enriched, while 12 MAM animals were scanned, six subsequently non-enriched and six subsequently enriched. For post enrichment measures, a subset of these animals were scanned again. Eight control animals were used, three control non-enriched (two males, one female), and five enriched (three males, two females), and 11 MAM animals, five non-enriched (two male, three female) and six enriched (three males, three females). 

**Figure 6:**
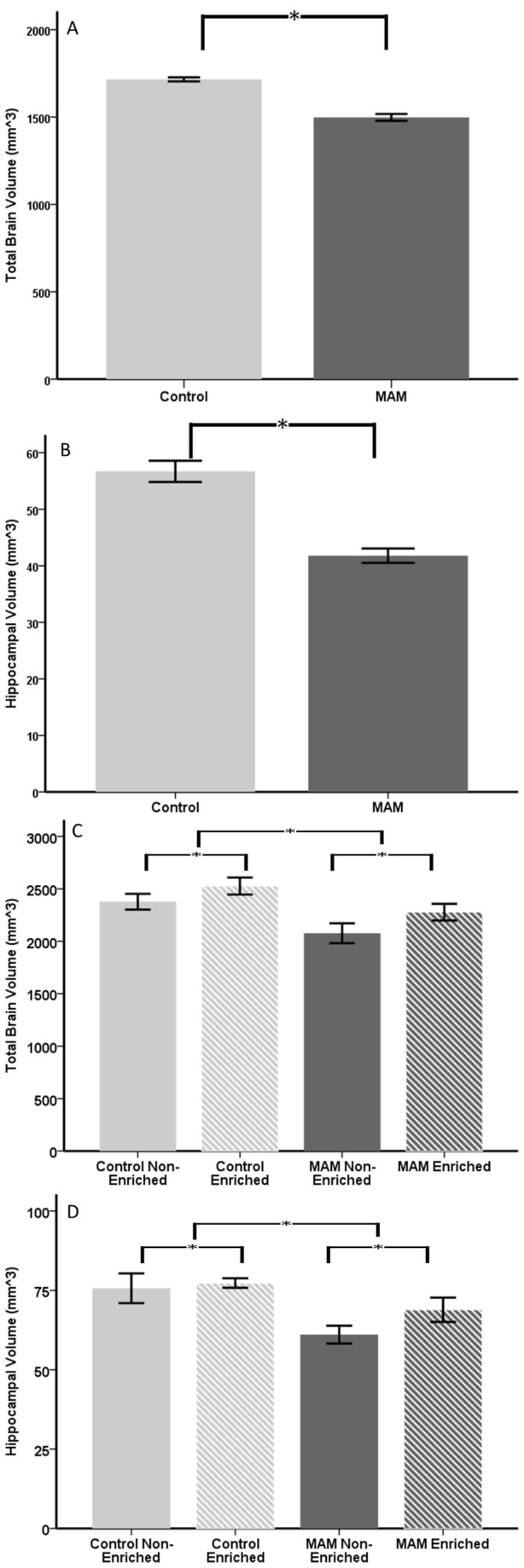
MRI data of MAM and control, enriched and non-enriched. At P22 MAM rats had significantly smaller total brain volume (**A**, p<0.001) and hippocampal volume (**B**, p<0.001). At P150, animals had undergone 128 days of either enrichment or non-enrichment. The effect of MAM was present in both total brain volume (**C**, p=0.015) and hippocampal volume (**D**, p<0.001). Enrichment increased total brain volume (**C**, p=0.024) and hippocampal volume (**D**, p=0.026) in both control and MAM animals. Error bars represent standard error.

In the pre enrichment scans, there was a significant effect of both gender (p<0.001) and MAM treatment (p<0.001) in total brain volume (Figure 6A). For hippocampal volume, there was a significant effect of both gender (p=0.004) and MAM treatment (p<0.001) (Figure 6B). There was no difference in baseline MRI between the animals that would later be enriched or non-enriched in either the MAM or control rat groups. 

For post enrichment, total brain volume was significantly less in MAM treated rats (p=0.015) than in control rats (Figure 6C). However, in both MAM and control animals the enriched animals had larger brains than the non-enriched ones (p=0.024). The total volume of the hippocampus was significantly smaller in MAM treated rats (p<0.001), and larger with enrichment (p=0.026) (Figure 6D). In both total brain volume and hippocampal brain volume, females were smaller than males (p<0.001 total brain volume, p=0.013 hippocampal brain volume).

**Figure 7:**
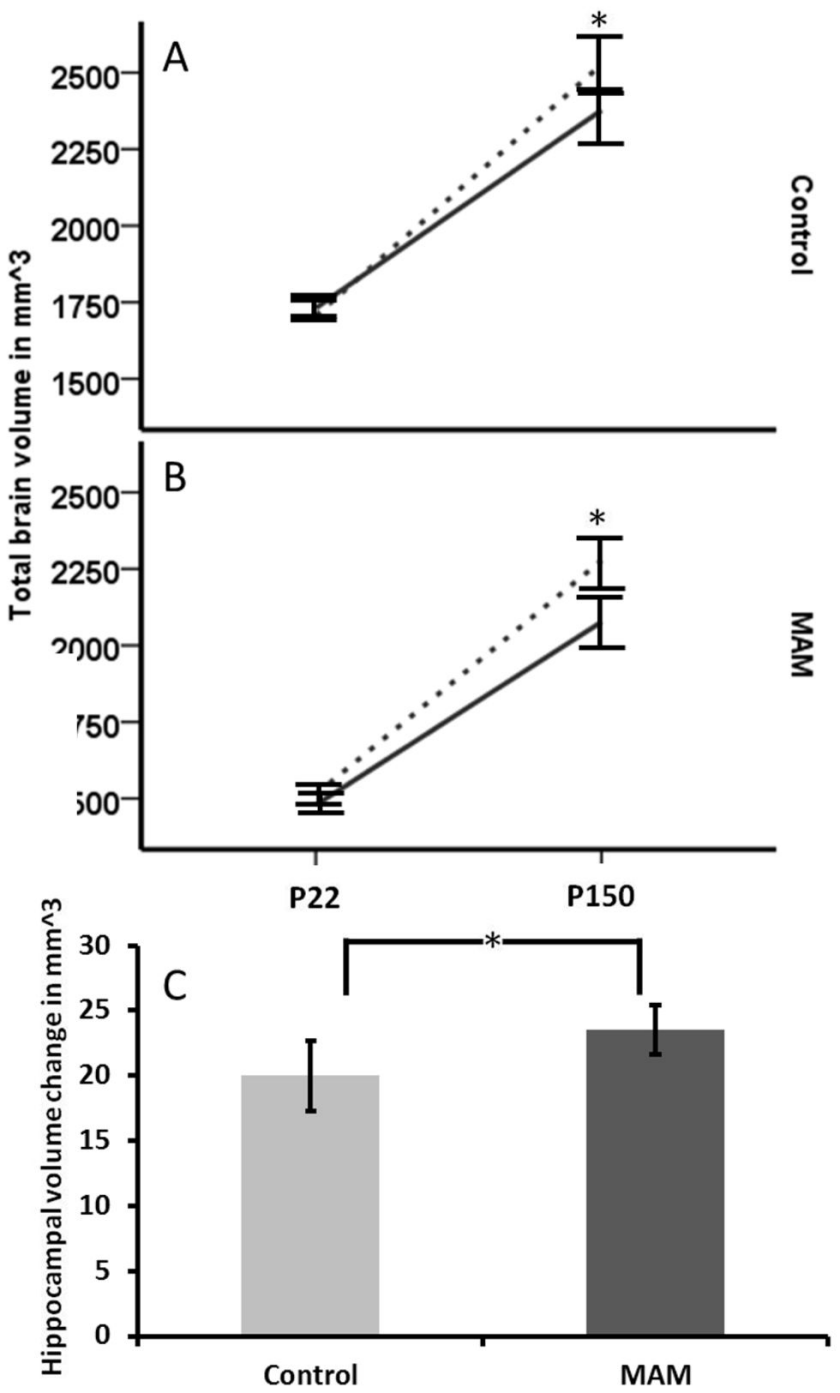
Longitudinal examination of whole brain and hippocampal growth from pre and post enrichment (or non-enrichment) MRI scans. (**A**) Control animals did not differ at the initial scan, but at P150 there was a significant difference between those housed in enriched vs. non-enriched settings (time*enrichment interaction; p=0.022). (**B**) The same was true for MAM animals. There was no enrichment effect in the hippocampal volumes (p=0.624). (**C**) For the hippocampus but not the whole brain, there was an effect of MAM on growth longitudinally (time*MAM interaction; p=0.009) as shown by a greater volume change over time in the MAM hippocampus compared to the controls. Error bars represent standard error.

In comparing the pre and post enrichment scans, we saw a time by enrichment interaction (p=0.022) but not a time by MAM interaction (p=0.862) (Figure 7A-B). This meant that the rate of brain volume growth was similar in control and MAM animals, but when comparing enriched and non-enriched animals the rate of growth was greater for the enriched animals. For hippocampal brain volumes, we did not show a time by enrichment interaction (p=0.624) but we did show a time by MAM interaction (p=0.009). This was a reflection of the greater hippocampal growth seen in MAM animals, with controls having increased hippocampal volume between the two time points by 27% while MAM increased hippocampal volume by 36% (Figure 7C). Despite the more rapid increase, the MAM hippocampus was 15% smaller than that of the controls on average. 

## Discussion

The results of these studies confirm that brains with a major structural abnormality have the anatomical and functional capacity to be cognitively enhanced through maximized environmental stimulus and increased training time. This finding is important as it suggests that such strategies may be effectively translated into human studies. We contend that children with MCD associated learning and behavioral impairments and seizures often have restrictions placed on their environments and educational access. Children may be prevented from participating in sports (e.g. biking, swimming), leisure activities (e.g. restricted access to television or computers) and often have days where they do not attend school. In addition to patients with MCD, schizophrenic patients often present with decreased medial temporal lobe volumes, as we see in the MAM model, and likely face similar developmental difficulties as children with MCD. Within this framework our environmental manipulation studies clearly suggest that a more deprived environment is associated with reductions in cognitive ability and brain volumes in both MAM and control animals. 

In the Morris water maze test we found that MAM animals took more time to find the platform than controls. Enrichment improved both MAM and control performance over their peers. What has been shown is that a spatial deficit, caused by a MCD, is partially alleviated simply by being housed with other rats and being exposed to objects rather than being isolated without objects. The level of achievement in these animals was limited below control levels, however there is room for growth and improvement through enrichment, and similarly there is a possibility of worsening impairment through isolation and non-enriched environments. Therefore, isolation of children with epilepsy associated with an MCD may be harmful to the child. In future trials we wish to further dissect this deficit in performance with variations on the Morris water maze, including a probe trial where the platform is removed or moved to a new location to assess strategy and cognitive flexibility respectively. 

The active place avoidance task requires a high level of attention and spatial awareness in order to avoid being shocked. In this test we eliminated enrichment, and chose to examine if we could train MAM animals to reach control levels of performance, or alternatively, if they could never attain the level of performance seen in control animals. In the learning phase, MAM animals took significantly more sessions to reach criterion than controls. They also had a decreased latency to first entrance, indicating impaired long term memory of the location of the shock zone from previous exposure. All control animals, after five sessions, reached criterion and were removed from the study. Continued training of the MAM animals showed a more gradual improvement in the number of entrances and shocks per session. After 15 sessions, all MAM animals reached criterion demonstrating that MAM animals could learn the location of a noxious stimulus and use spatial information to actively avoid this zone for a 10 minute trial. In setting criteria as less than five shocks in two consecutive sessions, we brought a long term memory component into the test, forcing animals to perform well not just for a single session, but two sessions separated temporally. This demonstrates that the MAM animals learn the task and retain this memory after removal from the testing space for several hours. 

Although improvement in hippocampal function is important, children with MCD often have behavioral or psychiatric disorders which are due to a cortical dysfunction. In order to investigate the impact of environmental manipulation on neocortical function we used the three chamber social choice test. Independent of MAM treatment, enrichment increased the time spent in the chamber with the rat, decreased the time spent in the chamber with the object, and increased the ratio of time spent in the chamber with the rat to time spent in the chamber with the object. This demonstrated that MAM rats with MCD show an increased likelihood to choose social novelty over exploration of a novel object if they were raised in a socially and environmentally enriched setting. However, animals were less likely to choose social novelty over object novelty when raised in deprived, isolated conditions. 

It is also interesting to note that MAM rats spent more time in the center chamber than controls. The rats were acclimated for five minutes to the center chamber prior to the start of the test, which decreased the likelihood that this could be explained by exploration of the center chamber. Rather, we could attribute this increased time in the center chamber to a lack of interest in exploring novelty or possible neophobia. Previous research on the MAM model showed that in the pretest phase of an object recognition task, when the rats were exposed to two objects for the first time prior to the subsequent recognition step, MAM rats spent less time exploring the objects than controls [20]. We concluded that MAM rats have an apparent deficit in exploring novelty in this paradigm, and that enrichment has no effect on this deficit. In future tests we would like to expand on this paradigm to include testing with a familiar and non-familiar rat to further examine the possibility of social deficits in MAM treated animals. 

MRI is a frequent diagnostic tool for physicians trying to confirm the presence of an MCD. In addition, while histology is a powerful tool it necessitates the sacrifice of the animal while MRI allowed us to longitudinally examine brain and hippocampal volume changes in the same animals. In our MRI analysis we showed that the effect of MAM treatment was detectable as a significant decrease in whole brain and hippocampal volumes. Enrichment also had an impact on whole brain volume and hippocampal volume, in both MAM and control animals. Control enriched brains were on average 1.06 times larger than control non-enriched brains and MAM enriched brains were 1.11 times larger than MAM non-enriched brains. This whole brain increase was remarkable, and a testament to the wide spanning impact of enrichment in the brain. 

When we examined the change in brain and hippocampal volume over time, we saw that MAM and controls did not differ in the change over time of their whole brain volume, but that the enriched animals showed an increased rate of change compared to non-enriched. With hippocampal volumes, enriched animals did not increase at a higher rate than non-enriched, but MAM animals did increase faster than controls. This may reflect a form of compensatory neurogenesis, as hypothesized in an E12 MAM exposure model [32], and a promising demonstration that the brain is plastic and imbued with the capacity to recover, at least in part, from early life damage. 

From our results we conclude that enrichment increases brain volume in a MCD model, and that enriched rats with MCD had corresponding improvements in the Morris water maze task and in sociability when compared to non-enriched counterparts. We also show that additional training will allow animals of this MCD model to behave at control levels in a spatial learning task. One possible confounding factor is that MAM treated animals were raised by mothers injected by MAM, which could lead to differences in maternal care. However, we are unable to find any literature supporting this view and we did not have concerns about maternal care when observing our groups. Given that MAM affects newly dividing cells for a limited time and given that there are few newly dividing cells in the mature brain we do not feel that this is an important factor in explaining our results.

Questions that remain to be addressed include whether enrichment started later in life may have similar effects as enrichment initiated post weaning, other behavioral paradigms that enrichment may improve that are relevant to human cognition and the molecular and more detailed anatomical changes that take place in MAM treated animals with enrichment. We would hope that the conclusion physicians and educators alike draw from these results are that: MCD and schizophrenic patients can benefit from both social and environmental exposure and suffer from restriction of the same, and that with repetitive training MCD and schizophrenic patients can learn tasks that they at first may struggle with when compared to others. Clinical studies have exposed children with autism to sensorimotor enrichment and seen encouraging improvements in cognition [33]. However, these children did not have MRI abnormalities. Our study gives encouraging evidence that enrichment strategies used in children with other cognitive struggles can be extended to children with cortical malformations. 
